# Amyloid positron emission tomography and cerebrospinal fluid results from a crenezumab anti-amyloid-beta antibody double-blind, placebo-controlled, randomized phase II study in mild-to-moderate Alzheimer’s disease (BLAZE)

**DOI:** 10.1186/s13195-018-0424-5

**Published:** 2018-09-19

**Authors:** Stephen Salloway, Lee A. Honigberg, William Cho, Michael Ward, Michel Friesenhahn, Flavia Brunstein, Angelica Quartino, David Clayton, Deborah Mortensen, Tobias Bittner, Carole Ho, Christina Rabe, Stephen P. Schauer, Kristin R. Wildsmith, Reina N. Fuji, Shehnaaz Suliman, Eric M. Reiman, Kewei Chen, Robert Paul

**Affiliations:** 1 Department of Neurology and Psychiatry, The Warren Alpert Medical School of Brown University, 345 Blackstone Boulevard, Providence, RI 2906 USA; 2 Genentech Inc., South San Francisco, CA USA; 3 F Hoffmann-La Roche Ltd., Basel, Switzerland; 4 Banner Alzheimer’s Institute, Phoenix, AZ USA

**Keywords:** Alzheimer’s disease, Biomarkers, Positron emission tomography, Monoclonal antibodies, Antibodies, Humanized

## Abstract

**Background:**

We investigated the effect of crenezumab, a humanized anti-amyloid-beta (Aβ) immunoglobulin (Ig)G4 monoclonal antibody, on biomarkers of amyloid pathology, neurodegeneration, and disease progression in patients with mild-to-moderate Alzheimer’s disease (AD).

**Methods:**

This double-blind, placebo-controlled, randomized phase II study enrolled patients with mild-to-moderate AD and a Mini-Mental State Examination (MMSE) score of 18–26. In part 1 of the study, patients were 2:1 randomized to receive low-dose subcutaneous (SC) 300 mg crenezumab every 2 weeks (q2w) or placebo for 68 weeks; in part 2, patients were 2:1 randomized to receive high-dose intravenous (IV) 15 mg/kg crenezumab every 4 weeks (q4w) or placebo for 68 weeks. The primary endpoint was change in amyloid burden from baseline to week 69 assessed by florbetapir positron emission tomography (PET) in the modified intent-to-treat population. Secondary endpoints were change from baseline to week 69 in cerebrospinal fluid (CSF) biomarkers and fluorodeoxyglucose PET, and change from baseline to week 73 in 12-point Alzheimer’s Disease Assessment Scale cognitive subscale (ADAS-Cog12) and Clinical Dementia Rating Sum of Boxes (CDR-SB). Safety was assessed in patients who received at least one dose of study treatment.

**Results:**

From August 2011 to September 2012, 91 patients were enrolled and randomized (low-dose SC cohort: crenezumab (*n* = 26) or placebo (*n* = 13); high-dose IV cohort: crenezumab (*n* = 36) or placebo (*n* = 16)). The primary endpoint was not met using a prespecified cerebellar reference region to calculate standard uptake value ratios (SUVRs) from florbetapir PET. Exploratory analyses using subcortical white matter reference regions showed nonsignificant trends toward slower accumulation of plaque amyloid in the high-dose IV cohort. In both cohorts, a significant mean increase from baseline in CSF Aβ(1–42) levels versus placebo was observed. Nonsignificant trends toward ADAS-Cog12 and CDR-SB benefits were identified in a mild (MMSE 20–26) subset of the high-dose IV cohort. No amyloid-related imaging abnormalities due to edema/effusion were observed.

**Conclusion:**

The primary endpoint was not met. Exploratory findings suggest potential Aβ target engagement with crenezumab and possible slower accumulation of plaque amyloid. Studies investigating the effects of higher doses of crenezumab on amyloid load and disease progression are ongoing.

**Trial registration:**

ClinicalTrials.gov, NCT01397578. Registered on 18 July 2011.

**Electronic supplementary material:**

The online version of this article (10.1186/s13195-018-0424-5) contains supplementary material, which is available to authorized users.

## Background

The deposition of extracellular insoluble amyloid plaques composed primarily of amyloid-beta (Aβ) peptides in the brain is a hallmark pathologic finding in Alzheimer’s disease (AD). However, it is hypothesized that the soluble oligomeric species of Aβ are the forms that are toxic to neurons and that lead to hyperphosphorylation and aggregation of tau [1]. This cascade of pathophysiologic events is thought to result in synaptic dysfunction, loss of neurons, and neurotransmitter deficits [2, 3]. Anti-amyloid therapies are designed to modify the course of the disease by targeting this cascade.

Crenezumab is a fully humanized immunoglobulin isotype G4 (IgG4) monoclonal antibody that binds with high affinity to Aβ oligomers while maintaining the ability to bind to other forms of Aβ (monomers, fibrils, and plaques) [4, 5]*.* The IgG4 backbone confers reduced binding of Fc-gamma receptors (FcγRs) compared with an IgG1 backbone, and was shown in vitro to preserve FcγR-mediated microglial phagocytosis and removal of oligomers while minimizing FcγR-mediated inflammatory activation of microglia and release of proinflammatory cytokines [4]. This reduced effector function of crenezumab is hypothesized to reduce cytokine-mediated neurotoxicity and reduce inflammation at sites of Aβ plaque deposition, particularly involving blood vessels. The latter was hypothesized to increase the risk of drug-induced amyloid-related imaging abnormalities (ARIA) in studies investigating anti-amyloid antibodies with full effector function [4].

This phase II, multicenter, randomized, double-blind, placebo-controlled, parallel-group study was designed to evaluate the effects of crenezumab on brain amyloid plaque load as assessed by florbetapir positron emission tomography (PET) and other biomarkers in patients with mild-to-moderate AD (ABE4955g, BLAZE; NCT01397578). The prespecified analysis of the florbetapir PET data used a cerebellar reference region for calculating standard uptake value ratios (SUVRs). Although this was the widely accepted reference region at the time this study was designed, more recent evidence published near the conclusion of this study showed that using a subcortical white matter reference region reduces longitudinal variability of SUVR [6–8]. Based on those findings, additional exploratory analyses of the florbetapir PET data were conducted using white matter reference regions that result in reduced variability in tracking longitudinal florbetapir SUVR changes. Other fluid and imaging biomarkers of AD were also evaluated in this study. BLAZE was independent from the phase II study, ABE4869g (ABBY; NCT01343966), which used the same treatment doses and duration but did not include amyloid PET imaging or mandatory cerebrospinal fluid (CSF) collection and focused on changes in cognition and functioning as endpoints [9].

## Methods

### Study design and participants

This study was conducted at 21 sites in the US, one site in Spain, and one site in France. The study protocol was approved by the respective institutional review boards prior to participant recruitment and was conducted in accordance with US Food and Drug Administration regulations, International Council on Harmonization E6 Guideline for Good Clinical Practice, and applicable local, state, federal, and country laws. Written informed consent was obtained from all patients prior to performing study-related procedures in accordance with federal and institutional guidelines. Patients, site staff, and sponsors were blinded to the treatment assignment and remained blinded, unless unblinding was recommended by the sponsor’s internal monitoring committee for the management of patient safety.

Patients were eligible to participate if they were 50–80 years of age, met the criteria for mild-to-moderate probable AD according to the National Institute of Neurologic and Communicative Disorders and Stroke/Alzheimer’s Disease and Related Disorders Association criteria, with Mini-Mental State Examination (MMSE) score of 18–26 points at the time of screening [10]. Additional inclusion criteria were a Geriatric Depression Scale (GDS-15) score of < 6, a Clinical Dementia Rating Sum of Boxes (CDR-SB) score of ≥ 0.5 [11, 12], and an Alzheimer’s Disease Assessment Scale cognitive subscale (ADAS-Cog) Delayed Word Recall score of ≥ 5 [13]. Patients were required to have evidence of elevated amyloid burden consistent with a diagnosis of AD indicating moderate-to-frequent neuritic plaques (Aβ-positive) as assessed by a central expert blinded visual reading of the screening florbetapir PET scan. Treatment with approved AD drugs such as acetylcholinesterase inhibitors (AChEIs) or memantine initiated ≥ 3 months and stabilized ≥ 2 months prior to randomization was permitted.

### Randomization and dosing

The study was conducted in two parts. In part 1, patients were randomly assigned 2:1 (crenezumab:placebo) to 300 mg subcutaneous (SC) crenezumab every 2 weeks (q2w) or placebo (low-dose SC cohort); in part 2, patients were randomly assigned 2:1 (crenezumab:placebo) to 15 mg/kg intravenous (IV) crenezumab every 4 weeks (q4w) or placebo (high-dose IV cohort). Part 2 of the study was initiated upon completion of recruitment for part 1. Both parts consisted of three periods: 1) a screening period (up to 42 days); 2) a treatment period (weeks 1–69); and 3) a safety follow-up period (weeks 70–85). Eligible patients that completed the week-73 assessment had the option to enroll in the open-label extension (OLE) study, GN28525, to evaluate the long-term safety and tolerability of crenezumab. All patients who enrolled in the OLE study received active drug at the same dosing frequency, dose level, and route of administration that they were assigned to in ABE4955g. Randomization was managed by a central IxRS vendor using dynamic hierarchical randomization, and in both parts of the study patients were randomized independently and stratified by apolipoprotein E ε4 (APOEε4) genotype (carrier vs noncarrier), MMSE score (< 22 vs ≥ 22), and study site. Site personnel were blinded to randomization details.

An acceptable safety and tolerability profile for crenezumab at the high dose (15 mg/kg q4w IV) was supported both by a phase I study, ABE4427g, and by results of a safety run-in assessment of ABE4869g, where no serious adverse events (SAEs) or dose-limiting toxicities were observed [9]. The high-dose IV cohort was estimated to provide approximately 2.5-times higher exposure (area under the curve (AUC)) compared with the low-dose SC cohort.

### Outcomes

The primary outcome measure was the change in brain amyloid burden from baseline to week 69, as assessed by florbetapir SUVRs using mean cortical and cerebellar gray matter reference regions. The secondary outcome measures were changes from baseline to week 69 in: 1) levels of Aβ(1–42), total tau (t-tau), and phosphorylated tau (p-tau) in the CSF; and 2) changes in fluorodeoxyglucose PET (FDG PET) measurements of cerebral-to-cerebellar glucose metabolism. Changes from baseline to week 73 in ADAS-Cog12 and CDR-SB were also measured. Subgroup analyses of the change from baseline to week 69 were performed to assess treatment differences in a mild (MMSE 20–26) patient subset. Additional exploratory outcome measures in which SUVR values were calculated using white matter reference regions, instead of cerebellar gray matter, were included as an alternative to the primary outcome measure due to the increased power of this approach and the longitudinal changes in cerebral glucose metabolism in an AD-related statistical region of interest (ROI; improved power demonstrated by these image analysis techniques in an independent longitudinal cohort, see below).

### CSF collection and analysis

CSF was collected from all patients at screening and prior to dosing at week 69 or at early termination/discontinuation (ET/D) if necessary. CSF (10–12 mL) was collected by lumbar puncture at L4/L5 with a Sprotte atraumatic needle into 15-mL low-retention polypropylene tubes (Sarstedt AG, Numbrecht, Germany), frozen immediately on dry ice, and transferred to −80 °C storage. For aliquoting, CSF was thawed on ice, vortexed for 30 s at maximum speed and centrifuged at 2000 g for 3 min. Aliquots (0.5 mL) were dispensed into 0.5-mL low-retention, screw-cap MAXYmum Recovery TM tubes using the corresponding low-retention pipette tips (Axygen Scientific Inc., Union City, CA) and frozen at −80 °C. CSF crenezumab concentrations were analyzed using a validated enzyme-linked immunosorbent assay (ELISA; limit of detection 12.5 ng/mL). Total (free and bound) CSF Aβ(1–42) was measured using the Elecsys® β-Amyloid(1–42) immunoassay under development by Roche Diagnostics (Penzberg, Germany) [14]. CSF t-tau and p-tau(181) were measured using INNOTEST® ELISAs (Fujirebio, Zwijnaarde, Belgium). CSF was stored at −80 °C and all samples were analyzed in batches using single reagent lots at the end of the study.

### Blood collection and analysis

Blood plasma was collected from all patients at screening and prior to dosing at weeks 1, 3 (SC dose only), 5, 13, 25, 49, 69, and 73, or at ET/D if necessary. Plasma Aβ(1–40) and Aβ(1–42) was measured using crenezumab-tolerant Elecsys® β-Amyloid(1–40) and Elecsys® β-Amyloid(1–42) immunoassays adapted for measurement in plasma by Roche Diagnostics. Plasma was stored at −80 °C and all samples were analyzed in batches using single reagent lots at the end of the study.

Blood samples were collected for crenezumab pharmacokinetic analyses from all patients at weeks 1, 2 (SC dose only), 3 (SC dose only), 5, 13, 25, 37, 49, 61, 69, and 73, or at ET/D. Serum crenezumab concentrations were analyzed using a validated ELISA (limit of detection 50 ng/mL).

### Magnetic resonance imaging acquisition and analysis

Magnetic resonance imaging (MRI) scans were performed during screening and then at weeks 7, 15, 23, 35, 47, 59, and 73, or at ET/D if necessary. The MRI protocol included the following scans for safety assessments and volumetric measurements: 1) a high-resolution T1-weighted structural scan; 2) a T2*-weighted gradient-recalled echo; and 3) a T2-weighted fluid-attenuated inversion recovery.

A central, blinded facility (NeuroRx, Montreal, QC, Canada) performed visual reads at all visits for safety assessments (see below), and made measurements of structural atrophy from the T1 images at baseline and weeks 23, 47, and 73. Bilateral volumes of the hippocampi and ventricles were measured using a method based on Anatomic Non-linear Image Matching And Labeling (ANIMAL) with a minimum deformation template library and local patch-based label fusion [15, 16]. The resulting volumes were visually inspected by experts and adjusted if necessary. The percentage change in whole-brain volume was measured with respect to baseline using SIENA [17], part of the Oxford Centre for Functional MRI of the Brain (FMRIB)’s Software Library [18].

### PET acquisition and analysis

Florbetapir PET scans were performed at screening (baseline) and week 47 and week 69 visits, or at ET/D if necessary. See Additional file 1 (Supplementary Methods) for more information about the image acquisition and analysis methods.

Two independent, blinded processing pipelines were used to measure composite cortical SUVR values for each scan. The SUVR is the ratio of the mean standard uptake value (SUV) of the composite neocortical region to the SUV of a reference region (as described below). The first pipeline, developed and executed by Molecular NeuroImaging (MNI; Molecular NeuroImaging LLC, New Haven, CT), was used for the predefined primary analysis and the post-hoc exploratory analysis. Baseline PET images were registered to the baseline T1 MRI. The transformation matrix derived from normalizing the MRI to standard space, T_MRI_, was applied to the PET images. Follow-up PET images were registered to the baseline MRI and then normalized by applying T_MRI_. Mean SUVs were extracted from regions of interest (ROIs) using an anatomical template that was individually refined by the gray matter mask segmented from the baseline MRI. The composite cortical ROI included the frontal cortex, parietal cortex, lateral temporal cortex, and posterior cingulate cortex (PCC). Two reference ROIs were used to calculate SUVR: 1) the cerebellar cortex, which was the predefined ROI for the primary outcome (SUVR_CB_); and 2) anterior bilateral volumes of subcortical white matter, which was measured as part of the initial analysis but only used for the post-hoc exploratory measurements (SUVR_MNI-WM_).

The second pipeline, a PET-only procedure developed and executed by Banner Alzheimer’s Institute (BAI; Phoenix, AZ) and recommended by Avid Radiopharmaceuticals (Philadelphia, PA) was used only for post-hoc exploratory analysis [6]. Baseline PET images were registered directly to a standard florbetapir PET template, which was also used to extract SUV measurements from anatomically defined ROIs [6]. The transformation matrix, T_PET_, was saved. Follow-up PET images were registered to the baseline PET and then normalized by applying T_PET_. The composite cortical ROI included the inferior medial frontal gyrus, superior parietal cortex, lateral temporal cortex, PCC, anterior cingulate cortex, and precuneus. The reference ROI was composed of white matter and included corpus callosum and centrum semiovale (SUVR_BAI-WM_).

FDG PET scans were performed at baseline and week 69 visits, or at ET/D if necessary. Images were processed using the same MNI pipeline described above. For SUVR measurements, the composite cortical ROI included the PCC, parietal cortex, and temporal cortex, and the reference ROI was the cerebellar cortex.

### Safety assessments

Safety was assessed based on reports of adverse events (AEs), SAEs, and adverse events of special interest (AESIs), which included ARIA-E (ARIA due to edema), ARIA-H (micro-hemorrhages and superficial siderosis), and macro-hemorrhages evident on MRI. Safety assessments also included clinical laboratory testing, vital signs, physical and neurologic examinations, prospective suicidality assessment, electrocardiography, and brain MRI. AEs were graded in severity using the National Cancer Institute Common Terminology Criteria for Adverse Events (NCI-CTCAE) grading scale. All safety data were assessed by an unblinded internal Safety Monitoring Committee on a regular basis. Blood samples were collected to test for the presence of anti-therapeutic antibody (ATA) in serum.

### Statistical analysis

Planned enrollment was approximately 36 patients into each dosing cohort (SC and IV). Assumptions of a 0.3-point difference in mean SUVR changes in florbetapir PET between crenezumab and placebo, a standard deviation (SD) across patients of 0.2 [19], and a 30% dropout rate yielded an estimated 90% confidence interval (CI) on the population SUVR treatment effect of approximately 0.18–0.42.

The primary analysis was performed on the modified intent-to-treat population, which was defined as all patients who were randomized and had both a baseline measurement and at least one postbaseline measurement for each endpoint. The analysis was conducted according to treatment assigned at randomization. The safety population included all patients who received at least one dose of study drug and was analyzed according to the treatment received (e.g., one patient was randomized to the placebo arm but received a single dose of crenezumab in error, and was therefore included in the crenezumab safety analysis).

The primary and secondary endpoints were analyzed as change scores (postbaseline value − baseline value). The baseline value was defined as the latest nonmissing predose value. Mean changes from baseline were compared across treatment arms using mixed modeling for repeated measures (MMRM) for endpoints with more than one postbaseline measurement (florbetapir PET, ADAS-Cog12, CDR-SB, and volumetric measurements from MRI), and using analysis of covariance (ANCOVA) for endpoints with only one postbaseline measurement (CSF Aβ(1–42), t-tau, p-tau, and FDG PET). Each ANCOVA and MMRM model had the following fixed effects: an intercept term, a term for baseline value of the endpoint, a term for APOEε4 strata (carrier vs noncarrier), a term for MMSE strata (< 22 vs ≥ 22), a term for APOEε4 strata by MMSE strata interaction, and a term for treatment arm. Each MMRM model had additional fixed effects: a categorical visit term and a term for treatment-by-visit interaction.

The respective models were used to estimate least squares mean changes from baseline within each treatment arm and visit, as well as mean differences between treatment arms (mean change in the placebo arm – mean change in the active arm) at each visit. Two-sided 95% CIs for the differences and associated two-sided unadjusted *p* values were also provided as an aid to interpretation of results for exploratory hypothesis generation. All analyses were performed using SAS Version 9.2.

## Results

### Study population

**Fig. 1:**
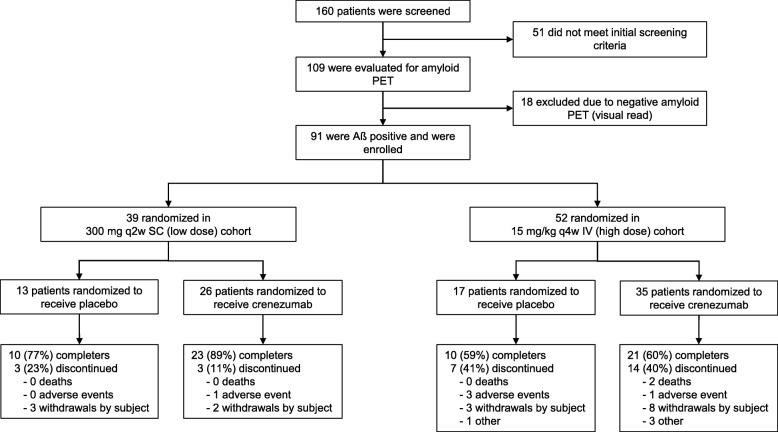
Study disposition. One patient randomized to placebo received one dose of crenezumab treatment and was therefore included in the crenezumab arm of the safety population. *IV* intravenous, *PET* positron emission tomography, *q2w* every 2 weeks, *q4w* every 4 weeks, *SC* subcutaneous

From August 2011 to September 2012, 160 patients were screened for the study (Fig. 1). Of these, 109 patients were eligible for screening with florbetapir PET, 18 (16.5%) of whom were assessed as Aβ-negative and were not included in the study. Of the Aβ-positive patients, 39 were randomized into the low-dose SC cohort (placebo, *n* = 13; crenezumab, *n* = 26), and 52 into the high-dose IV cohort (placebo, *n* = 17; crenezumab, *n* = 35). One patient in the high-dose IV cohort was randomized to the placebo arm but received a single dose of crenezumab in error, and was therefore included in the crenezumab arm of the safety population. As a result, the safety population for the high-dose IV cohort was *n* = 16 for placebo and *n* = 36 for crenezumab.

### Baseline characteristics

Demographic characteristics, including age, race, ethnicity, and body weight at baseline, were generally well balanced between the placebo and crenezumab treatment arms (Table 1). More females were included than males (57.1% vs 42.9%), particularly in the crenezumab treatment arms (62.3% vs 37.7%, respectively).

**Table 1:** Baseline characteristics (randomized population)

	Low-dose SC cohort (*n* = 39)		High-dose IV cohort (*n* = 52)	
	Placebo (*n* = 13)	Crenezumab (*n* = 26)	Placebo (*n* = 17)	Crenezumab (*n* = 35)
Age (years), mean (SD)	68.9 (8.3)	66.7 (9.5)	69.8 (7.7)	71.4 (7.1)
Sex, female (%)	61.5	53.8	35.3	68.6
MMSE score, mean (SD)	22.3 (2.4)	21.5 (2.4)	20.5 (2.2)	20.8 (2.3)
MMSE 20–26 (mild), %	92.3	73.1	58.8	60.0
APOEε4 carriers, %	92.3	84.6	70.6	68.6
ADAS-Cog12 score, mean (SD)	28.9 (7.4)	29.4 (9.7)	34.5 (11.1)	31.2 (9.9)
CDR-SB score, mean (SD)	4.3 (1.5)	4.2 (2.1)	5.9 (1.9)	4.9 (2.0)
ADCS-ADL score, mean (SD)	66.8 (6.8)	65.4 (9.5)	64.5 (8.2)	66.8 (7.4)
SUVR (cerebellar gray reference)	1.9 (0.3)	1.8 (0.3)	1.8 (0.3)	1.7 (0.3)
AChEI and/or memantine use, %	92.3	84.6	82.4	91.4

*AChEI* acetylcholinesterase inhibitors, *ADAS-Cog12* 12-point Alzheimer’s Disease Assessment Scale cognitive subscale, *ADCS-ADL* Alzheimer’s Disease Cooperative Study Activities of Daily Living, *ApoE* apolipoprotein E, *CDR-SB* Clinical Dementia Rating Sum of Boxes, *IV* intravenous, *mITT* modified intent-to-treat population, *MMSE* Mini-Mental State Examination, *SC* subcutaneous, *SD* standard deviation, *SUVR* standard uptake value ratio

APOEε4 carriers (E4^+^) accounted for 76.9% of the evaluable population (70 of 91 randomized patients). The ratio of carriers to noncarriers (E4^−^) was higher in the low-dose SC cohort (87.2% E4^+^) relative to the high-dose IV cohort (69.6% E4^+^), but was generally well balanced between placebo and crenezumab treatment arms within each cohort. The mean baseline MMSE score at screening was 22 for the low-dose SC cohort and 21 for the high-dose IV cohort, and was comparable between placebo and treatment arms.

In total, 64 patients (70.3%) completed the week 69 amyloid PET and had biomarker assessments at week 69 and clinical assessments at week 73. A total of 27 patients (29.7%) discontinued treatment. The most frequent reasons for treatment discontinuation were withdrawal by subject (17.6%, 16 patients) followed by discontinuation due to an AE (5.5%, five patients), with no observed cluster or pattern of AE.

Use of other approved AD therapies was balanced between placebo and crenezumab treatment arms in both the low-dose SC and high-dose IV cohorts. Overall, 46.7% of placebo- and 44.0% of crenezumab-treated patients received AChEIs only, while 36.7% of placebo- and 41.0% of crenezumab-treated patients received combined treatment with AChEIs and memantine.

### Primary analysis

**Fig. 2:**
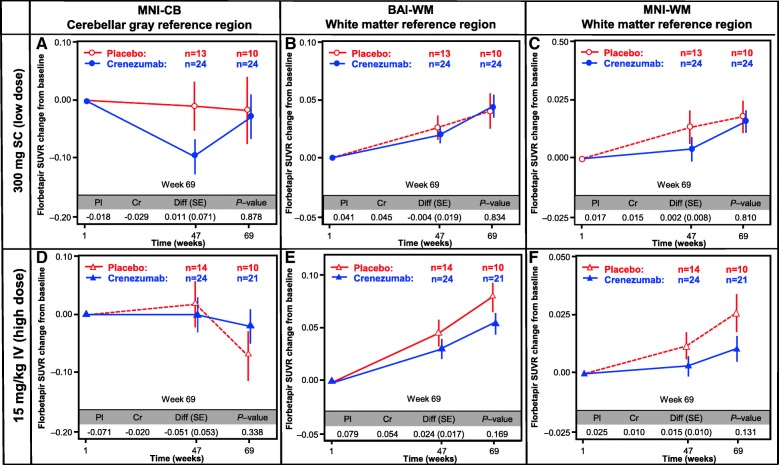
Amyloid PET analysis. Analysis of the florbetapir change from baseline using three different methods for the calculation of SUVR: in the cerebellar gray MNI-CB (**a,d**), BAI-WM (**b,e**), and MNI-WM (**c,f**). The primary difference between these methods is the choice of reference region: cerebellar gray matter (SUVR_MNI-CB_) or subcortical white matter (SUVR_MNI-WM_ and SUVR_BAI-WM_). The reference regions in both the low-dose SC (**a–c**) and high-dose IV (**d–f**) cohorts are shown. *BAI* Banner Alzheimer’s Institute, *BL* baseline, *Cr *crenezumab, *Diff* difference, *IV* intravenous, *MNI* molecular neuroimaging, *Pl* placebo, *SC* subcutaneous, *SE* standard error, *SUVR* standard uptake value ratio, *WM* white matter, *CB* cerebellar

There was no evidence of a treatment effect in either the low-dose SC or the high-dose IV cohort for the predefined primary endpoint: change in SUVR_CB_ (florbetapir PET) from baseline to week 69 (Fig. 2a, d). Plots comparing SUVR at baseline versus week 69 for individual patients indicated greater than expected longitudinal variability using the cerebellar reference region, including many placebo patients with apparent lowering in florbetapir SUVRs at week 69 (Additional file 2: Figure S1A, D).

### Secondary analyses

Using the exploratory analyses with white matter reference regions (SUVR_BAI-WM_ and SUVR_MNI-WM_), the longitudinal variability observed in the primary analysis was reduced, and fewer placebo patients showed evidence of amyloid reduction (Additional file 2: Figure S1B, C, E, F). There was a nonsignificant trend toward treatment difference observed in the high-dose IV cohort of 0.024 (95% CI –0.011 to 0.060; unadjusted *p* = 0.169) for SUVR_BAI-WM_, representing a 30% reduction in amyloid accumulation relative to placebo (Fig. 2e). Similarly, in the high-dose IV cohort, a nonsignificant trend toward treatment difference of 0.015 (95% CI –0.005 to 0.035; unadjusted *p* = 0.131) was observed for SUVR_MNI-WM_, representing a 60% reduction in amyloid accumulation relative to placebo (Fig. 2f). No treatment effect was observed in the low-dose SC cohort for SUVR_MNI-WM_ and SUVR_BAI-WM_.

No significant treatment effects were observed for any of the MRI-derived volumetric changes (hippocampus, ventricles, whole brain) from baseline to week 73 in either the low-dose SC or high-dose IV cohorts (Additional file 3: Figure S2). There was also no significant treatment effect observed in mean FDG PET SUVR changes from baseline to week 69 in either cohort (Additional file 4: Figure S3).

### Safety

Overall, and similar to observations in the larger crenezumab phase II study ABE4869g [9], crenezumab was well tolerated in this study with similar rates of AEs across the combined treatment arms (placebo 96.6%; crenezumab 93.5%) and SAEs (placebo 13.8%; crenezumab 14.5%). SAEs were generally balanced between the low-dose SC (placebo: 7.7%; crenezumab: 11.5%) and high-dose IV cohorts (placebo: 18.8%; crenezumab: 16.7%; Table 2). Two patients (2.2%) treated with crenezumab developed pneumonia (one in each dose cohort). Two deaths occurred in the crenezumab arm, both in the high-dose IV cohort (Table 2). Neither death was considered by investigators to be related to the study drug. One death resulted from pleural effusion leading to respiratory failure, while the other was sudden death of unknown etiology. No deaths occurred in the placebo arm.

**Table 2:** Summary of adverse events

	Low-dose SC cohort (*n* = 39)		High-dose IV cohort (*n* = 52)		All patients (*n* = 91)	
	Placebo (*n* = 13)	Crenezumab (*n* = 26)	Placebo (*n* = 16)	Crenezumab (*n* = 36)	Placebo (*n* = 29)	Crenezumab (*n* = 62)
Patients with ≥ 1 AE	13 (100)	26 (100)	15 (93.8)	32 (88.9)	28 (96.6)	58 (93.5)
Death	0 (0.0)	0 (0.0)	0 (0.0)	2 (5.6)*	0 (0.0)	2 (3.2)*
SAEs	1 (7.7)	3 (11.5)	3 (18.8)	6 (16.7)	4 (13.8)	9 (14.5)
AEs of grade ≥ 3	1 (7.7)	4 (15.4)	4 (25.0)	7 (19.4)	5 (17.2)	11 (17.7)
Pneumonia	0 (0.0)	1 (3.8)	0 (0.0)	1 (2.8)	0 (0.0)	2 (3.2)
Most frequent AEs						
Back pain	2 (15.4)	5 (19.2)	2 (12.5)	5 (13.9)	4 (13.8)	10 (16.1)
Upper respiratory tract infection	0 (0.0)	7 (26.9)	2 (12.5)	4 (11.1)	2 (6.9)	11 (17.7)
Anxiety	0 (0.0)	9 (34.6)	0 (0.0)	3 (8.3)	0 (0.0)	12 (19.4)
Nasopharyngitis	4 (30.8)	4 (15.4)	13 (18.8)	1 (2.8)	7 (24.1)	5 (8.1)
Depression	2 (15.4)	5 (19.2)	2 (12.5)	2 (5.6)	4 (13.8)	7 (11.3)

Further details can be found in the Additional file 1 (Supplementary safety summary)

All values are shown as *n* (%)

*AE* adverse event, *IV* intravenous, *SAE* serious adverse event; *SC* subcutaneous

*Neither fatal event was assessed as related to study drug by the investigator

The most frequent AESI was the development of ARIA-H, which was reported in 3.4% (one patient) of placebo patients versus 14.5% (nine patients) of those receiving crenezumab (Additional file 5: Table S1). All cases of ARIA-H documented during the study were asymptomatic and classified as NCI-CTCAE Grade 1 AEs in all but one case, the latter being NCI-CTCAE Grade 2 severity. All patients with ARIA-H were able to continue study treatment except for one placebo-assigned patient diagnosed with superficial siderosis who discontinued from treatment as stipulated in the protocol. No cases of ARIA-E were documented during the study (Table 2, Additional file 5: Table S1, and Additional file 1: Supplementary Safety Summary).

### Exploratory CSF and plasma biomarker analyses

**Fig. 3:**
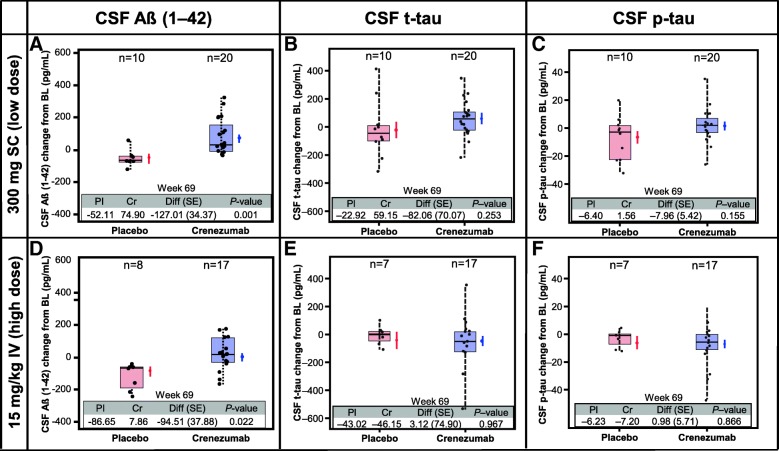
CSF biomarkers. Analysis of the change in biomarker levels found in CSF (Aβ(1–42) (**a,d**), t-tau (**b,e**), and p-tau (**c,f**)) in both the low-dose SC (**a**–**c**) and high-dose IV (**d**–**f**) cohorts. CSF t-tau and p-tau was not analyzed for one patient at week 69. *BL* baseline, *Cr* crenezumab, *CSF* cerebrospinal fluid, *Diff* difference, *IV* intravenous, *Pl* placebo, *p-tau* phosphorylated tau, *SC* subcutaneous, *SE* standard error

CSF concentrations of Aβ(1–42) were assessed in 55 patients, and CSF t-tau and p-tau concentrations assessed in 54 patients, at baseline and week 69 (Fig. 3). There was a significant increase in CSF Aβ(1–42) concentrations in patients treated with crenezumab. In the low-dose SC cohort, CSF Aβ(1–42) mean change from baseline was −52.11 pg/mL in the placebo arm, while in the crenezumab arm mean change from baseline was +74.90 pg/mL (crenezumab vs. placebo difference of 127.01 pg/mL, *p* = 0.001). In the high-dose IV cohort, CSF Aβ(1–42) mean change from baseline was −86.65 pg/mL in the placebo arm, whereas in the crenezumab arm mean change from baseline was +7.86 pg/mL (crenezumab vs. placebo difference of 94.51 pg/mL, *p* = 0.022). There was no evidence of a treatment difference in mean change from baseline for CSF t-tau or p-tau between crenezumab and placebo for either the low-dose SC or the high-dose IV cohort (Fig. 3b, c, e, f). No apparent correlation was observed between time-matched crenezumab pharmacokinetics and Aβ(1–42) changes in CSF (Additional file 6: Figure S4).

**Fig. 4:**
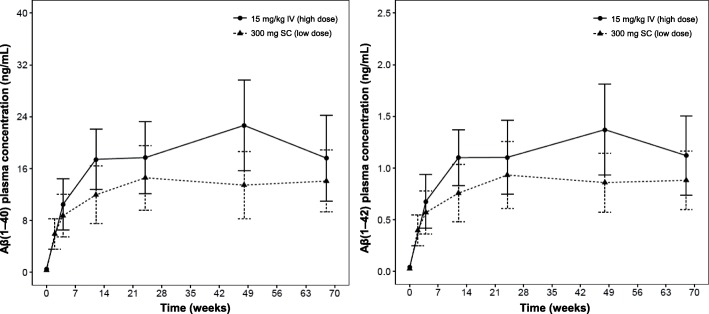
Aβ(1–40) and Aβ(1–42) plasma concentrations. Mean (±SD) Aβ(1–40) and Aβ(1–42) plasma concentrations following low-dose SC or high-dose IV administration to patients with mild-to-moderate AD (weeks 1–69). *Aβ* beta-amyloid, *AD* Alzheimer’s Disease, *IV* intravenous, *SC* subcutaneous, *SD* standard deviation

Total plasma Aβ(1–40) concentrations increased from 0.327 ng/mL to 14.1 ng/mL and from 0.562 ng/mL to 17.6 ng/mL after 68 weeks of treatment with crenezumab in the low-dose SC and high-dose IV cohorts, respectively (Fig. 4). Similarly, total plasma Aβ(1–42) concentrations increased from 0.027 ng/mL to 0.882 ng/mL and from 0.041 ng/mL to 1.12 ng/mL after 68 weeks of treatment with crenezumab in the low-dose SC and high-dose IV cohorts, respectively (Fig. 4). This indicates an overall increase in plasma Aβ(1–40) and Aβ(1–42) concentrations in patients treated with crenezumab but not in patients treated with placebo.

### Exploratory cognitive analyses

**Fig. 5:**
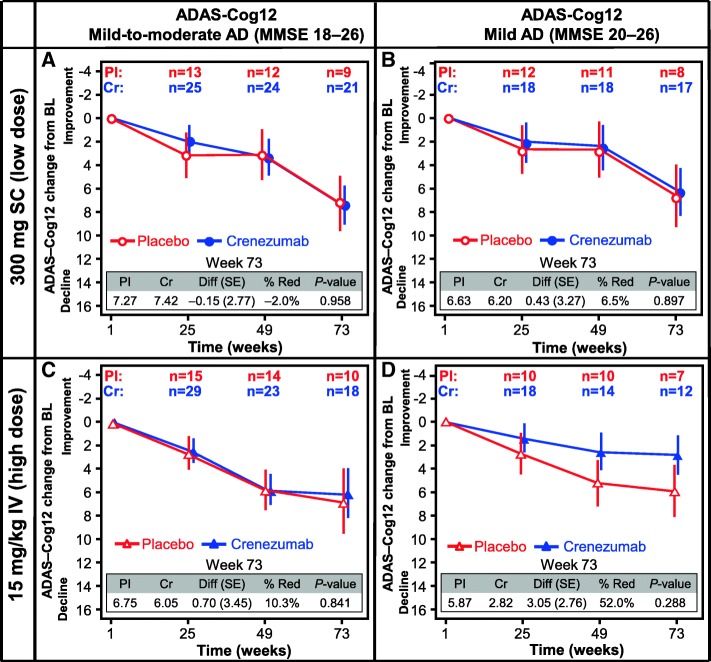
ADAS-Cog12. Change from baseline of ADAS-Cog12 score in patients with mild-to-moderate AD (**a,c**) or mild AD (**b,d**) in both the low-dose SC (**a,b**) and the high-dose IV (**c,d**) cohort. *% Red* percentage reduction, *AD* Alzheimer’s disease, *ADAS-Cog12 12-point* Alzheimer’s Disease Assessment Scale cognitive subscale, *BL* baseline, *Cr* crenezumab, *Diff *difference, *IV *intravenous, *MMSE* Mini-Mental State Examination, *Pl* placebo, *SC* subcutaneous, *SE* standard error

Exploratory analyses of the change from baseline to week 73 in ADAS-Cog12 (Fig. 5a, c) and CDR-SB (Additional file 7: Figure S5A, C) did not show a treatment effect in either the low-dose SC or high-dose IV cohorts. However, in a subset of mild AD patients (MMSE 20–26) in the high-dose IV cohort, a reduction in decline in ADAS-Cog12 (52.0%; ADAS-Cog12 difference 3.05 points; 95% CI –2.90 to 9.01; unadjusted *p* = 0.288) and CDR-SB (41.5%; CDR-SB difference 0.80 points; 95% CI –1.31 to 2.91; unadjusted *p* = 0.439) from baseline to week 73 was observed in the crenezumab arm relative to placebo, although this was not significant. No treatment effect was observed for the mild AD subset in the low-dose SC cohort (Fig. 5b, d and Additional file 7: Figure S5B, D).

## Discussion

In this small 68-week proof-of-concept study, crenezumab failed to demonstrate a significant impact on changes in PET measurements of amyloid burden using cerebral-to-cerebellar florbetapir SUVRs, our original primary endpoint. However, an exploratory analysis found a nonsignificant trend toward reduced cerebral-to-white matter SUVRs, a measurement with increased power to track longitudinal change in amyloid burden, in the high-dose IV-treated cohort. The possibility that higher-dose crenezumab treatment may be associated with reduced fibrillar Aβ accumulation will be tested in ongoing phase III studies.

Treatment effects were not observed for the other secondary endpoints of changes in FDG PET, CSF t-tau, and p-tau measurements. No treatment effect was observed in the clinical outcomes, ADAS-Cog12 and CDR-SB, in the overall study population, but in the mild subset (MMSE 20–26), a nonsignificant trend toward a clinical benefit was observed in the high-dose IV cohort, which was consistent with observations in the recently published phase II ABE4869g study [9], suggesting that greater benefits for patients may be possible by treating earlier disease stages and with higher doses of crenezumab.

Initial reports that used amyloid PET in clinical trials of amyloid-targeted therapeutics used cerebellar reference regions to calculate SUVR [19–21], and similar methods have been used in more recent trials [22–24]. This reference region has been widely used based on the evidence from early cross-sectional studies that showed how SUVR could be used to quantitatively differentiate groups of individuals with different levels of cognitive impairment, but more recent reports analyzing the longitudinal florbetapir PET data from the Alzheimer’s Disease Neuroimaging Initiative (ADNI) have demonstrated that reference regions containing subcortical white matter can reduce variability in the measurement of SUVR changes over time [6–8, 25]. Evidence of these improvements was first published near the time that ABE4955g was completing and prompted the initiation of two additional blinded analyses performed with processing pipelines that had been independently optimized and validated using ADNI data [6, 7]. Although these alternative methods (SUVR_MNI-WM_ and SUVR_BAI-WM_) used different white matter references and different image processing techniques, both demonstrated: 1) more consistent amyloid accumulation in the placebo arm; 2) reduced longitudinal variability across treatment arms; and 3) nonsignificant trends in reductions of amyloid accumulation in the high-dose IV crenezumab-treated cohort compared with placebo. Further justification for using subcortical white matter as an improved reference region for longitudinal florbetapir PET measurements has been shown in exploratory analyses of other therapeutic studies with aducanumab and solanezumab [26, 27].

Aducanumab and gantenerumab have reported reductions in amyloid SUVR using cerebellar reference regions [22, 24], but these antibodies bind amyloid plaque and have full immune effector function; thus, they may be acting by a direct plaque removal mechanism effecting greater changes in the amyloid PET signal. The results of this study appear consistent with the hypothesis that crenezumab may reduce the further accumulation of amyloid plaque by enabling the removal of precursor soluble oligomers, even if it does not dramatically clear existing plaques. Detecting this subtle effect on longitudinal plaque accumulation may have required the more sensitive white-matter reference methods.

Treatment with crenezumab at both dose levels was associated with an increase in CSF Aβ(1–42), providing evidence for target engagement in the central nervous system, albeit not direct evidence of target engagement in the brain. Increases in CSF Aβ(1–42) relative to placebo were also observed in both dose groups in the ABE4869g trial [9], providing further evidence of target engagement. CSF samples were collected at trough (prior to final dose) when there was only a 1.7-fold mean difference in crenezumab levels between the SC and IV cohorts, and this may explain why similar increases in CSF Aβ(1–42) were observed at both dose levels. The increase in CSF Aβ(1–42) associated with crenezumab treatment could reflect increased input of Aβ(1–42) into the CSF from the brain or periphery, decreased clearance of CSF Aβ(1–42) out of the CSF, and/or a shift in CSF Aβ content toward more species that are detected by the Aβ(1–42) assay; thus, this observation does not definitively indicate a reversal of AD pathophysiology. Crenezumab treatment was also associated with a dose-dependent increase in plasma levels of Aβ(1–42) and Aβ(1–40). Plasma increases in Aβ have also been observed with solanezumab [28] and are thought to reflect an increase in the half-life of circulating Aβ when bound to antibody. Additional studies are needed to better understand the effects of crenezumab on plasma Aβ, CSF Aβ, and amyloid PET, and the relationship of these pharmacodynamic effects to the underlying pathology and cognitive decline.

Disease progression as evaluated using the cognitive and global measures ADAS-Cog12 and CDR-SB showed no treatment effect in the overall study population. However, in a subset of patients with milder disease (MMSE 22–26), a nonsignificant trend toward a treatment effect was observed in the high-dose IV group but not in the low-dose SC group. These data are consistent with results from the ABE4869g study which showed increasing treatment effect in ADAS-Cog12 in progressively milder subsets of AD patients [9]. Together, these two phase II studies suggest that higher doses of crenezumab may be needed to impact clinical outcomes.

No treatment effect on CSF t-tau or p-tau, or on FDG PET, was associated with crenezumab at either dose level. A clear relationship between changes in biomarkers of AD and clinical benefit is yet to be established in therapeutic studies [29, 30]. Discrepancies between biomarker and cognitive outcomes have been observed in trials of other anti-amyloid compounds, including bapineuzumab, gantenerumab, aducanumab, and solanezumab. In phase III studies in mild-to-moderate AD, bapineuzumab stabilized or slightly reduced (< 5%) amyloid plaque as measured by ^11^C-labeled Pittsburgh Compound-B PET but no clinical benefit was observed [20]. In a phase Ib study in prodromal-to-mild AD, aducanumab reduced amyloid load by as much as 19% at 1 year at the highest dose, and a dose-response relationship was observed for both amyloid removal and clinical benefit [22]. In a phase III study in prodromal AD, gantenerumab resulted in a 5.8% mean reduction in amyloid plaque levels at week 60 [24]. In that study, cognitive benefit compared with patients treated with placebo was not observed [24], but there was evidence of efficacy in patients with faster disease progression who had higher drug exposure [31]. Recent data on gantenerumab, as well as phase IIb data from BAN2401, have confirmed that higher doses of full-effector function anti-Aβ antibodies can result in more robust effects on amyloid plaque removal such that a substantial number of patients have their amyloid burden reduced to below the threshold for amyloid positivity within 1–2 years [32, 33]. Cognitive benefit was reported for BAN2401 [33] but this awaits confirmation in a larger study. In contrast, the monomer-only binding antibody solanezumab demonstrated no significant effect on amyloid plaque [34] and, although the phase III solanezumab studies did not meet their primary endpoint (ADAS-Cog14), a small but consistent effect on other cognitive and functional endpoints was detected in patients with mild AD [23]. Overall, modest (< 10%) reductions in CSF t-tau and p-tau have been observed in some studies [24, 35], but CSF results are not available for all anti-Aβ trials and thus far there is no consistent relationship between changes in CSF tau and changes in cognition. Given the discrepancies between the biomarker data and the clinical outcome across anti-Aβ studies, replication in larger studies will be required to understand further the relationship between biomarkers and clinical benefit.

Overall, crenezumab was well tolerated at the doses investigated in a mild-to-moderate AD (MMSE 18–26) population. No cases of ARIA-E were observed in ABE4955g, and only one asymptomatic case was observed in ABE4869g at the higher dose (a sulcal effusion in the right occipital, parietal, and temporal regions and in the left occipital region, reported at week 23 MRI assessment, in a 72-year-old APOEε4 homozygous female patient treated with crenezumab IV) [9]. The low levels of ARIA-E observed in both ABE4869g and ABE4955g enable evaluation of crenezumab at higher dose levels compared with anti-amyloid monoclonal IgG1 antibodies with full effector functions.

The limitations of this study include the small sample size and the absence of testing of doses > 15 mg/kg, which limits the power of the study to exclude a significant treatment effect on clinical and biomarker endpoints. One phase Ib (GN29632; NCT02353598) and two phase III studies investigating crenezumab in an earlier AD patient population are ongoing (BN29552, CREAD: NCT02670083; BN29553, CREAD2: NCT03114657). GN29632 and BN29553 will further explore the potential effects of crenezumab on cognition, biomarkers, and safety in prodromal-to-mild AD (MMSE 22–30) at monthly doses of 60 mg/kg IV.

## Conclusion

Here we report the results of the phase II ABE4955g trial evaluating the impact of crenezumab on brain amyloid load and related biomarkers in mild-to-moderate AD with evidence of brain amyloid pathology based on amyloid PET. The primary endpoint was not met. Exploratory analyses evaluated two alternative methods of analyzing longitudinal amyloid PET data using different reference regions. These methods reduced longitudinal variability and suggested a possible reduction in amyloid accumulation in crenezumab-treated patients. The trial also found a nonsignificant trend toward reduced cognitive decline in crenezumab-treated mild AD patients that was also observed in the ABE4869g study, suggesting that earlier treatment and higher doses may be associated with improved clinical outcome. Given the favorable safety profile of crenezumab, administration of an increased dose regimen in early AD patients in future studies may be possible to increase efficacy, without a corresponding increase in risk to patient safety. The two phase III CREAD studies (BN29552 and BN29553) for crenezumab are underway to test this hypothesis.

## Additional files

Additional file 1: Supplementary methods and safety summary. PET imaging parameters and summary of safety findings. (PDF 39 kb)

Additional file 2: **Figure S1.** SUVR longitudinal analysis. SUVR at week 69 compared to SUVR at baseline using different processing methods and reference regions: MNI-CB (A and D), BAI-WM (B and E), and MNI-WM (C and F) in both the low-dose SC (A–C) and high-dose IV (D –F) cohorts. (PPTX 157 kb)

Additional file 3: **Figure S2.** vMRI. Analysis of vMRI change from baseline in the hippocampus (A and B), ventricular volume (C and D), and whole brain (E and F) in low-dose SC (A, C, and E) and high-dose IV cohorts (B, D, and F). (PDF 98 kb)

Additional file 4: **Figure S3.** FDG PET. FDG PET SUVR change from baseline in the low-dose SC (A) and high-dose IV (B) cohorts. (PDF 65 kb)

Additional file 5: **Table S1.** Summary of ARIA events. Summary of ARIA events in the low-dose SC cohort, high-dose IV cohort, and all patients. (PDF 40 kb)

Additional file 6: **Figure S4.** CSF Aβ(1–42) crenezumab correlation analysis. Correlation analysis of change in CSF Aβ(1–42) from baseline and crenezumab concentrations at week 69 in patients in the low-dose SC cohort (circles) and high-dose cohort (triangles). (PDF 98 kb)

Additional file 7: **Figure S5.** CDR-SB. Change from baseline (BL) of CDR-SB score in patients with mild-to-moderate AD (A and C) or mild AD (B and D) in the low-dose SC (A and B) and high-dose IV (C and D) cohorts. (PDF 112 kb)

## Abbreviations

Aβ: Amyloid-beta
AChEI: Acetylcholinesterase inhibitor
AD: Alzheimer’s disease
ADAS-Cog: Alzheimer’s Disease Assessment Scale cognitive subscale
ADNI: Alzheimer’s Disease Neuroimaging Initiative
AE: Adverse event
AESI: Adverse event of special interest
ANCOVA: Analysis of covariance
APOEε4: Apolipoprotein E ε4
ARIA: Amyloid-related imaging abnormalities
ARIA-E: Amyloid-related imaging abnormalities representing vasogenic edema
ARIA-H: Amyloid-related imaging abnormalities representing microhemorrhage and siderosis
ATA: Anti-therapeutic antibody
CDR-SB: Clinical Dementia Rating Sum of Boxes
CI: Confidence interval
CSF: Cerebrospinal fluid
ELISA: Enzyme-linked immunosorbent assay
ET/D: Early termination/discontinuation
FcγR: Fc-gamma receptor
FDG: Fluorodeoxyglucose
IgG1: Immunoglobulin isotype G1
IgG4: Immunoglobulin isotype G4
IV: Intravenous
MMRM: Mixed modeling for repeated measures
MMSE: Mini-Mental State Examination
MRI: Magnetic resonance imaging
NCI-CTCAE: National Cancer Institute Common Terminology Criteria for Adverse Events
OLE: Open-label extension
PCC: Posterior cingulate cortex
PET: Positron emission tomography
p-tau: Phosphorylated tau
q2w: Every 2 weeks
q4w: Every 4 weeks
ROI: Region of interest
SAE: Serious adverse event
SC: Subcutaneous
SUV: Standard uptake value
SUVR: Standard uptake value ratio
t-tau: Total tau
