# Supplementary material for: Amyloid positron emission tomography and cerebrospinal fluid results from a crenezumab anti-amyloid-beta antibody double-blind, placebo-controlled, randomized phase II study in mild-to-moderate Alzheimer’s disease (BLAZE)
Source: Alzheimers Res Ther. 2018 Sep 19;10:96. doi: 10.1186/s13195-018-0424-5 (PMC6146627; doi:10.1186/s13195-018-0424-5)
Supplement: Supplementary file 1 — Supplementary methods and safety summary. PET imaging parameters and summary of safety findings. (PDF 39 kb) [file 13195_2018_424_MOESM1_ESM.pdf]

Supplementary Methods:

<u>PET Imaging Parameters:</u> Florbetapir PET Images were acquired starting 50 min after injection of florbetapir (10 mCi). Scan duration was either: 1) 30 min (6 x 5-minute dynamic frames), or 2) 15 min (single static frame) on systems that did not support dynamic scanning. Nominal scanning and reconstruction parameters were: 128 x 128 matrix; 600 mm field-of-view (4.7 x 4.7 mm in-plane resolution); 4 mm slice thickness; zoom factor of 2; 5 mm Gaussian filter; and corrections for random events, scatter, and decay. FDG PET images were acquired 30 min after injection of FDG (10 mCi). The scan duration, scan parameters, and reconstruction parameters were the same as described for the florbetapir PET scans above.

Supplementary Safety Summary:

The numbers of patients who reported any AE were generally similar in the two treatment arms (placebo: 96.6%, 28 patients; crenezumab: 93.5%, 58 patients). SAEs were reported in 13 patients (14.3%), and were balanced between the placebo (four patients, 13.8%) and treatment arms (nine patients, 14.5%). SAEs were also balanced between the low-dose SC (placebo: one patient, 7.7%; crenezumab: three patients, 11.5%) and high-dose IV cohorts (placebo: three patients, 18.8%; crenezumab: six patients, 16.7%). Two patients (2.2%) developed pneumonia (both received crenezumab treatment). No deaths were reported among patients in the placebo arm, while there were two (3.2%) fatal events among patients in the crenezumab arm, both in the IV cohort. Neither death was considered by investigators to be related to study drug. One death resulted from pleural effusion leading to respiratory failure, while the other was sudden death of unknown etiology.

The rates of injection- or infusion-related localized AEs were not balanced across treatment arms, with more reports among patients in the crenezumab arm than in the placebo arm (27.4% vs 13.8%, respectively). The most frequent injection- or infusion-related events were injection- or infusion-site extravasation in the IV cohort, and injection-site erythema or

swelling in the low-dose SC cohort. All reports of injection- or infusion-related localized AEs were of mild or moderate intensity (NCI-CTCAE Grade 1 or 2), except for one non-serious report of severe injection-site pain in a patient receiving low-dose SC crenezumab treatment. No events were reported as serious, and anaphylaxis or serious hypersensitivity reactions were not reported in either treatment arm.

The most frequent AESI was the development of ARIA-H, which was reported in one placebo patient (3.4%) versus nine crenezumab patients (14.5%, 4 in low-dose cohort and 5 in high-dose cohort) (Supplementary Table 1). All cases of ARIA-H were asymptomatic and were NCI-CTCAE Grade 1 AEs in all but one case (NCI-CTCAE Grade 2). All patients with ARIA-H were able to continue study treatment, except for one placebo patient diagnosed with superficial siderosis, who discontinued from treatment as stipulated in the protocol. No ARIA-E was documented throughout the study.

There was no observed development of post-baseline ATAs in either placebo- or crenezumab-treated patients, and there were no clinically significant findings related to vital signs or laboratory test results, or echocardiograms (ECGs).
