# Supplementary material for: Amyloid positron emission tomography and cerebrospinal fluid results from a crenezumab anti-amyloid-beta antibody double-blind, placebo-controlled, randomized phase II study in mild-to-moderate Alzheimer’s disease (BLAZE)
Source: Alzheimers Res Ther. 2018 Sep 19;10:96. doi: 10.1186/s13195-018-0424-5 (PMC6146627; doi:10.1186/s13195-018-0424-5)
Supplement: Supplementary file 2 — Figure S1. SUVR longitudinal analysis. SUVR at week 69 compared to SUVR at baseline using different processing methods and reference regions: MNI-CB (A and D), BAI-WM (B and E), and MNI-WM (C and F) in both the low-dose SC (A–C) and high-dose IV (D –F) cohorts. (PPTX 157 kb) [file 13195_2018_424_MOESM2_ESM.pptx]

## Slide 1
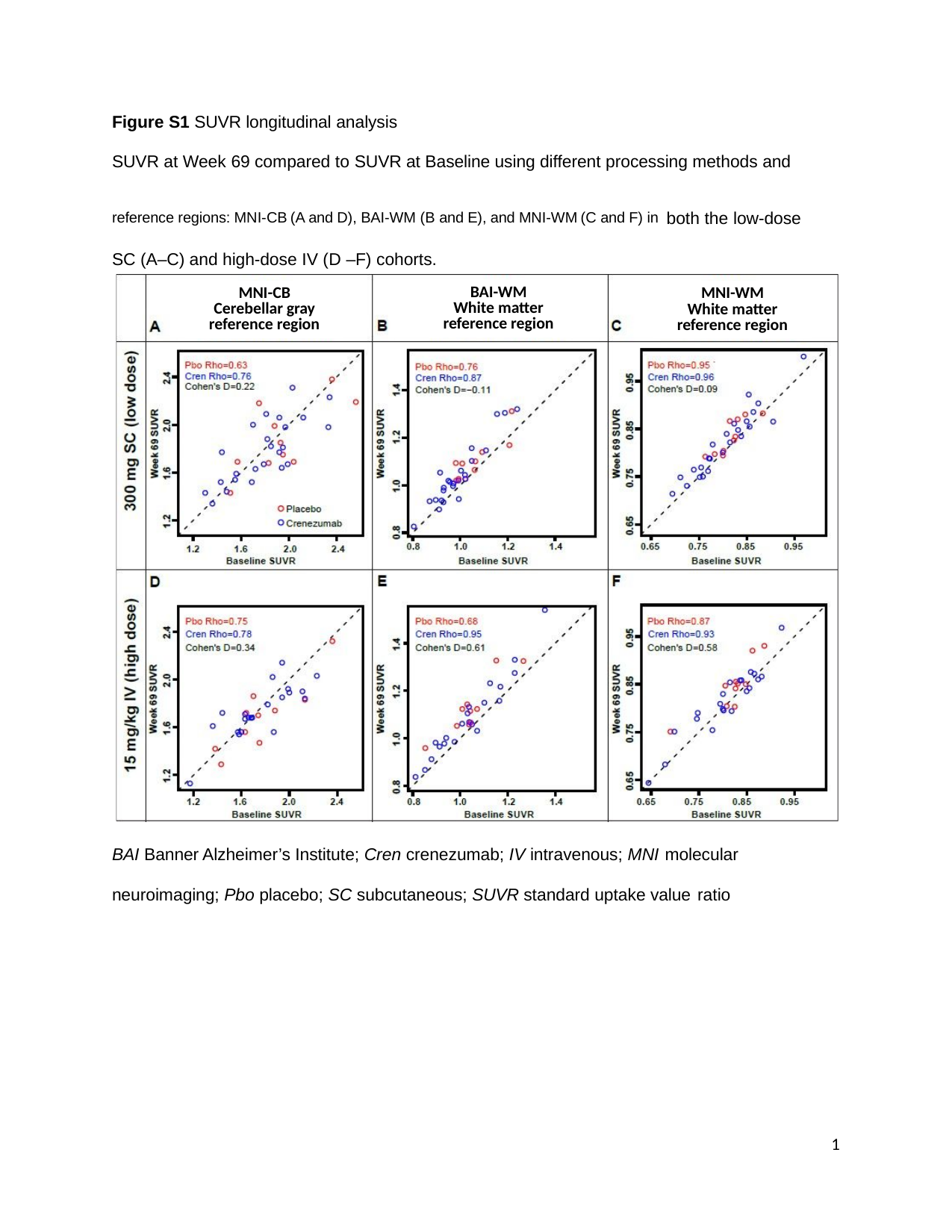

Figure S1 SUVR longitudinal analysis
SUVR at Week 69 compared to SUVR at Baseline using different processing methods and reference regions: MNI-CB (A and D), BAI-WM (B and E), and MNI-WM (C and F) in both the low-dose SC (A–C) and high-dose IV (D –F) cohorts.
BAI-WM
White matter
reference region
MNI-CB
Cerebellar gray
reference region
MNI-WM
White matter
reference region
BAI Banner Alzheimer’s Institute; Cren crenezumab; IV intravenous; MNI molecular
neuroimaging; Pbo placebo; SC subcutaneous; SUVR standard uptake value ratio
1
