# Supplementary material for: Amyloid positron emission tomography and cerebrospinal fluid results from a crenezumab anti-amyloid-beta antibody double-blind, placebo-controlled, randomized phase II study in mild-to-moderate Alzheimer’s disease (BLAZE)
Source: Alzheimers Res Ther. 2018 Sep 19;10:96. doi: 10.1186/s13195-018-0424-5 (PMC6146627; doi:10.1186/s13195-018-0424-5)
Supplement: Supplementary file 3 — Figure S2. vMRI. Analysis of vMRI change from baseline in the hippocampus (A and B), ventricular volume (C and D), and whole brain (E and F) in low-dose SC (A, C, and E) and high-dose IV cohorts (B, D, and F). (PDF 98 kb) [file 13195_2018_424_MOESM3_ESM.pdf]

Fig. S2 vMRI

Analysis of vMRI change from baseline in the hippocampus (panels A and B), ventricular volume (C and D), and whole brain (E and F) in low-dose SC (A, C, and E) and high-dose IV cohorts (B, D, and F).

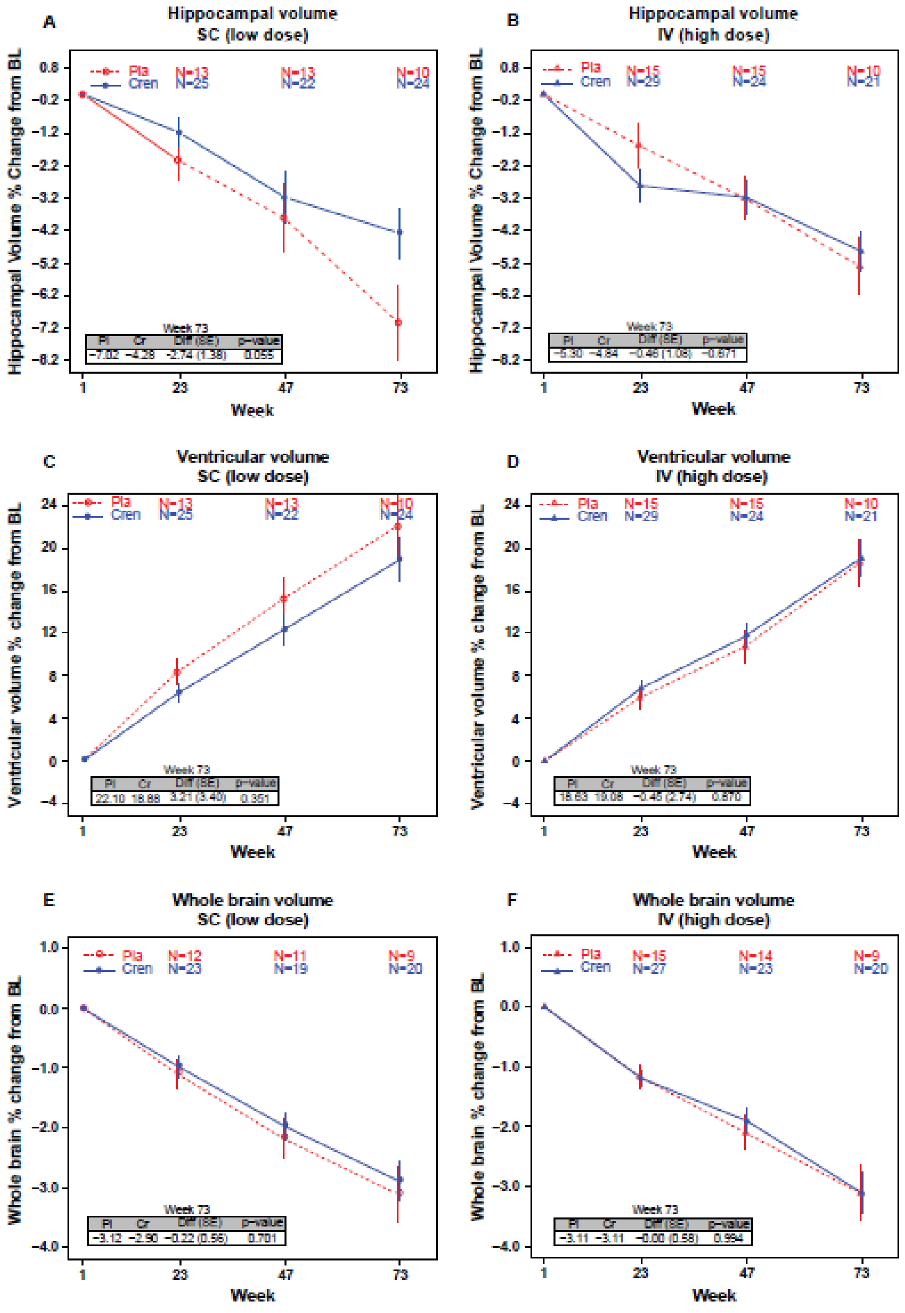

BL baseline; *Cr/Cren* crenezumab; *Diff* difference; *IV* intravenous; *MMSE* Mini-Mental State Examination; *Pl/Pla* placebo; *SC* subcutaneous; *SE* standard error; *vMRI* volumetric magnetic resonance imaging
