# Supplementary material for: Amyloid positron emission tomography and cerebrospinal fluid results from a crenezumab anti-amyloid-beta antibody double-blind, placebo-controlled, randomized phase II study in mild-to-moderate Alzheimer’s disease (BLAZE)
Source: Alzheimers Res Ther. 2018 Sep 19;10:96. doi: 10.1186/s13195-018-0424-5 (PMC6146627; doi:10.1186/s13195-018-0424-5)
Supplement: Supplementary file 4 — Figure S3. FDG PET. FDG PET SUVR change from baseline in the low-dose SC (A) and high-dose IV (B) cohorts. (PDF 65 kb) [file 13195_2018_424_MOESM4_ESM.pdf]

Fig. S3 FDG PET

FDG PET SUVR change from baseline in the low-dose SC (A) and high-dose IV (B) cohorts.

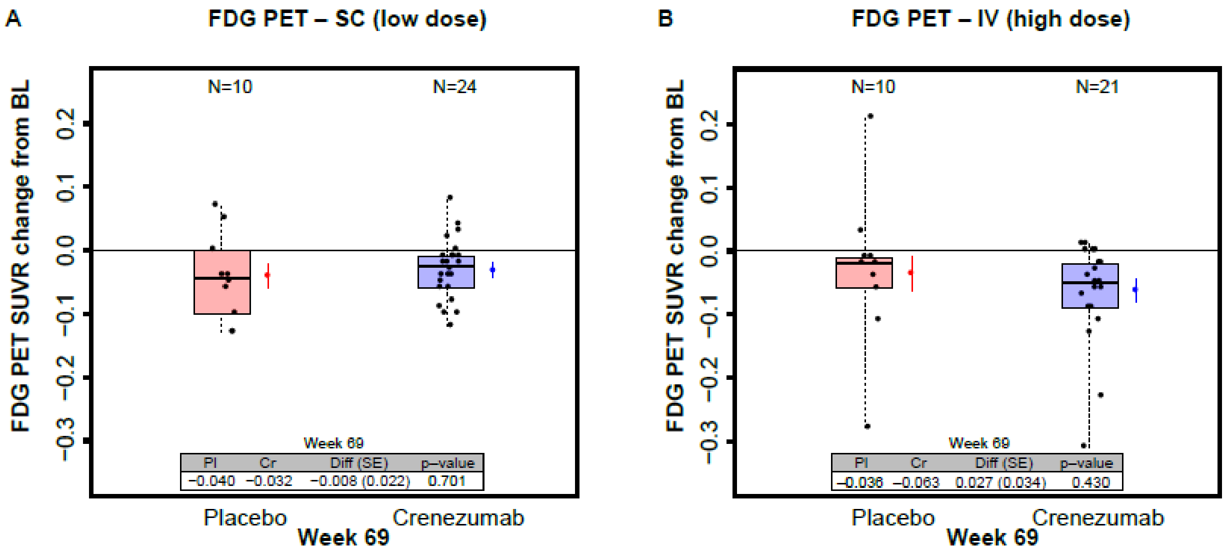

Cr crenezumab; *Diff* difference; *FDG* fluorodeoxyglucose (^{18}F); *IV* intravenous; *PET* positron emission tomography; *Pl* placebo; *SC* subcutaneous; *SE* standard error
