# Supplementary material for: Amyloid positron emission tomography and cerebrospinal fluid results from a crenezumab anti-amyloid-beta antibody double-blind, placebo-controlled, randomized phase II study in mild-to-moderate Alzheimer’s disease (BLAZE)
Source: Alzheimers Res Ther. 2018 Sep 19;10:96. doi: 10.1186/s13195-018-0424-5 (PMC6146627; doi:10.1186/s13195-018-0424-5)
Supplement: Supplementary file 5 — Table S1. Summary of ARIA events. Summary of ARIA events in the low-dose SC cohort, high-dose IV cohort, and all patients. (PDF 40 kb) [file 13195_2018_424_MOESM5_ESM.pdf]

Table S1 Summary of ARIA events

	Low-dose SC cohort (*n* = 39)		High-dose IV cohort (*n* = 52)		All patients (*n* = 91)	
	Placebo (*n* = 13)	Crenezumab (*n* = 26)	Placebo (*n* = 16)	Crenezumab (*n* = 36)	Placebo (*n* = 29)	Crenezumab (*n* = 62)
Any ARIA-E, n (%)						
Symptomatic	0 (0.0)	0 (0.0)	0 (0.0)	0 (0.0)	0 (0.0)	0 (0.0)
Asymptomatic	0 (0.0)	0 (0.0)	0 (0.0)	0 (0.0)	0 (0.0)	0 (0.0)
Superficial siderosis, n (%)	0 (0.0)	0 (0.0)	1 (6.3)	0 (0.0)	1 (3.4)	0 (0.0)
Macrohemorrhage, n (%)	0 (0.0)	0 (0.0)	0 (0.0)	0 (0.0)	0 (0.0)	0 (0.0)
New ARIA-H, n (%)						
Symptomatic	0 (0.0)	0 (0.0)	0 (0.0)	0 (0.0)	0 (0.0)	0 (0.0)
Asymptomatic	0 (0.0)	4 (15.4)	1 (6.3)	5 (13.9)	1 (3.4)	9 (14.5)
1 microhemorrhage	0 (0.0)	1 (3.8)	0 (0.0)	1 (2.8)	0 (0.0)	2 (3.2)
2–4 microhemorrhages	0 (0.0)	2 (7.7)	1 (6.3)	2 (5.6)	1 (3.4)	4 (6.5)
>4 microhemorrhages	0 (0.0)	1 (3.8)	0 (0.0)	2 (5.6)	0 (0.0)	3 (4.8)

ARIA-E amyloid-related imaging abnormalities: vasogenic edema or effusions; *ARIA-H* amyloid-related imaging abnormalities: microhemorrhage and siderosis; *IV* intravenous; *SC* subcutaneous
