# Supplementary material for: Amyloid positron emission tomography and cerebrospinal fluid results from a crenezumab anti-amyloid-beta antibody double-blind, placebo-controlled, randomized phase II study in mild-to-moderate Alzheimer’s disease (BLAZE)
Source: Alzheimers Res Ther. 2018 Sep 19;10:96. doi: 10.1186/s13195-018-0424-5 (PMC6146627; doi:10.1186/s13195-018-0424-5)
Supplement: Supplementary file 6 — Figure S4. CSF Aβ(1–42) crenezumab correlation analysis. Correlation analysis of change in CSF Aβ(1–42) from baseline and crenezumab concentrations at week 69 in patients in the low-dose SC cohort (circles) and high-dose cohort (triangles). (PDF 98 kb) [file 13195_2018_424_MOESM6_ESM.pdf]

Fig. S4 CSF Aβ(1–42) crenezumab correlation analysis

Correlation analysis of change in CSF Aβ(1–42) from baseline and crenezumab concentrations at Week 69 in patients in the low-dose SC cohort (circles) and high-dose cohort (triangles).

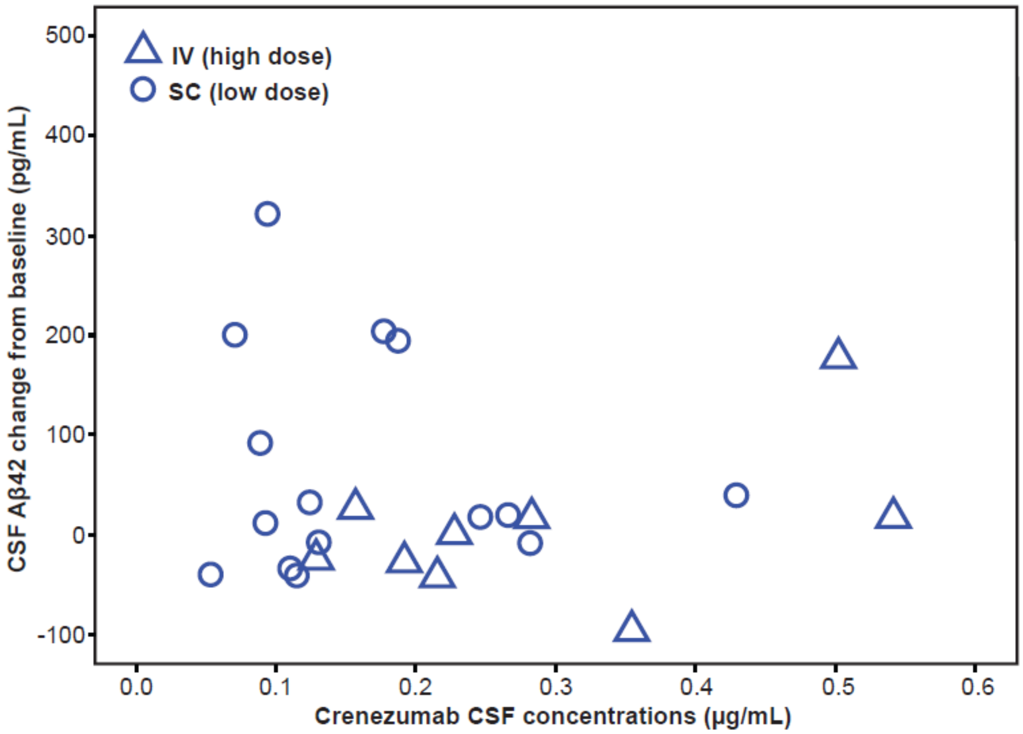

Aβ amyloid-beta; *CSF* cerebrospinal fluid; *IV* intravenous; *SC* subcutaneous
