# Supplementary material for: Amyloid positron emission tomography and cerebrospinal fluid results from a crenezumab anti-amyloid-beta antibody double-blind, placebo-controlled, randomized phase II study in mild-to-moderate Alzheimer’s disease (BLAZE)
Source: Alzheimers Res Ther. 2018 Sep 19;10:96. doi: 10.1186/s13195-018-0424-5 (PMC6146627; doi:10.1186/s13195-018-0424-5)
Supplement: Supplementary file 7 — Figure S5. CDR-SB. Change from baseline (BL) of CDR-SB score in patients with mild-to-moderate AD (A and C) or mild AD (B and D) in the low-dose SC (A and B) and high-dose IV (C and D) cohorts. (PDF 112 kb) [file 13195_2018_424_MOESM7_ESM.pdf]

Fig. S5 CDR-SB

Change from baseline (BL) of CDR-SB score in patients with mild-to-moderate AD (A and C) or mild AD (B and D) in the low-dose SC (A and B) and high-dose IV (C and D) cohorts.

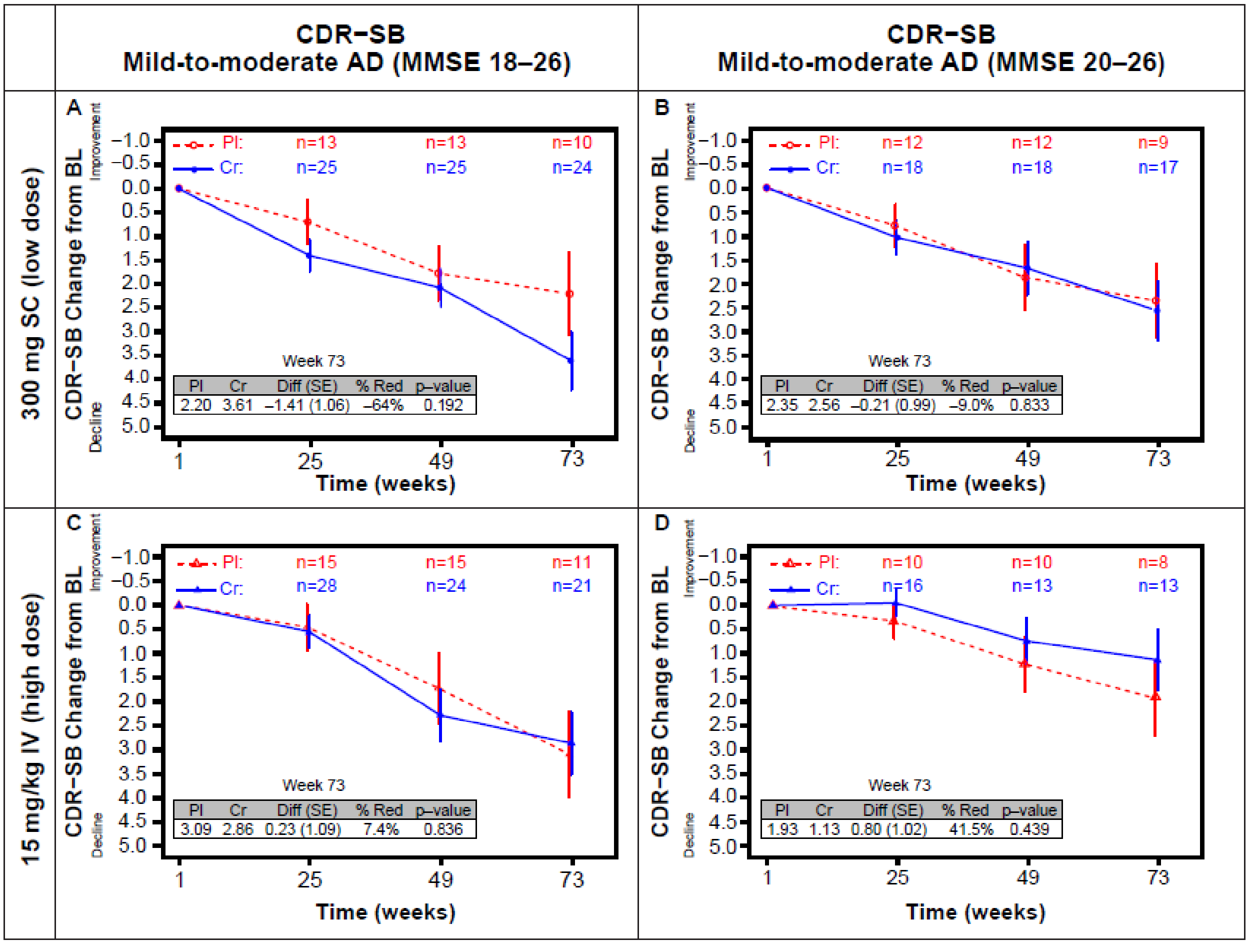

% Red percentage reduction; *AD* Alzheimer's disease; *BL* baseline; *CDR-SB* Clinical Dementia Rating-Sum of Boxes; *Cr* crenezumab; *Diff* difference; *IV* intravenous; *MMSE* Mini-Mental State Examination; *Pl* placebo; *SC* subcutaneous; *SE* standard error
